# Natural Reno-Protective Agents against Cyclosporine A-Induced Nephrotoxicity: An Overview

**DOI:** 10.3390/molecules27227771

**Published:** 2022-11-11

**Authors:** Sabrin R. M. Ibrahim, Hossam M. Abdallah, Ali M. El-Halawany, Gamal A. Mohamed, Aisha A. Alhaddad, Waad A. Samman, Ali A. Alqarni, Akaber T. Rizq, Kholoud F. Ghazawi, Riham Salah El-Dine

**Affiliations:** 1 Preparatory Year Program, Department of Chemistry, Batterjee Medical College, Jeddah 21442, Saudi Arabia; 2 Department of Pharmacognosy, Faculty of Pharmacy, Assiut University, Assiut 71526, Egypt; 3 Department of Natural Products and Alternative Medicine, Faculty of Pharmacy, King Abdulaziz University, Jeddah 21589, Saudi Arabia; 4 Department of Pharmacognosy, Faculty of Pharmacy, Cairo University, Cairo 11562, Egypt; 5 Department of Pharmacology and Toxicology, College of Pharmacy, Taibah University, Al-Madinah Al-Munawwarah 30078, Saudi Arabia; 6 Pharmaceutical Care Department, Ministry of National Guard—Health Affairs, Jeddah 22384, Saudi Arabia; 7 Clinical Pharmacy Department, College of Pharmacy, Umm Al-Qura University, Makkah 24382, Saudi Arabia

**Keywords:** cyclosporine A, natural products, nephrotoxicity, phenolics, reno-protective

## Abstract

CA (cyclosporine A) is a powerful immunosuppressing agent that is commonly utilized for treating various autoimmune illnesses and in transplantation surgery. However, its usage has been significantly restricted because of its unwanted effects, including nephrotoxicity. The pathophysiology of CA-induced kidney injury involves inflammation, apoptosis, tubular injury, oxidative stress, and vascular injury. Despite the fact that exact mechanism accountable for CA’s effects is inadequately understood, ROS (reactive oxygen species) involvement has been widely proposed. At present, there are no efficient methods or drugs for treating CA-caused kidney damage. It is noteworthy that diverse natural products have been investigated both in vivo and in-vitro for their possible preventive potential in CA-produced nephrotoxicity. Various extracts and natural metabolites have been found to possess a remarkable potential for restoring CA-produced renal damage and oxidative stress alterations via their anti-apoptosis, anti-inflammatory, and antioxidative potentials. The present article reviews the reported studies that assess the protective capacity of natural products, as well as dietary regimens, in relation to CA-induced nephrotoxicity. Thus, the present study presents novel ideas for designing and developing more efficient prophylactic or remedial strategies versus CA passive influences.

## 1. Introduction

The kidneys are vital organs that play an important role in removing waste and toxic materials from the blood, maintain electrolyte balance, and regulate the homeostasis of blood plasma, blood volume, blood pressure, and red blood cell genesis. The kidneys can be damaged or may become totally inactive due to several factors, such as oxidative stress, inflammation, ischemia/reperfusion injury, diabetes, and nephrotoxic agents, most often various modern drugs that are in use at present [1]. Just as some natural products are a source of substances that protect the kidneys, there are several plants known for their harmful effects on the kidneys, leading to renal dysfunction, ranging from acute to chronic renal failure and death. The herbal medicines mostly associated with nephrotoxicity are: *Dioscorea quinqueloba*, *Lawsonia inermis*, *Cassia senna* L., *Artemisia herba-alba*, *Chenopodium polyspermum*, Cape aloes, *Euphorbia paralias*, *Crataegus orientalis*, *Colchicum autumnale,* and *Tribulus terrestris* [2]. Additionally, in Persian medicine, a total of 64 plants that cause kidney damage have been reported, out of which *Allium schoenoprasum* and *Marrubium vulgare* were the most common nephrotoxic plants, but without relevant scientific evidence. Asafetida, garlic, saffron, and wormwood have been reported for their therapeutic effects on the kidneys, in addition to their kidney-damaging potential. Meanwhile, *Cymbopogon citratus*, *Amaranthus* spp., and *Artemisia absinthium* have been known to cause a direct nephrotoxic effect [3]

Nephrotoxicity is the condition in which the kidneys cannot properly detoxify and excrete drugs and toxic chemicals due to their destruction or damage caused by endo- or exogenous toxicants [4]. This is distinguished by increasing serum creatinine and urea and reducing the rate of GFR (glomerular filtration), and may be accompanied by arterial hypertension. Histologically, renal pathological changes occur, such as the swelling of the tubular cells, necrosis, arterial changes, and interstitial fibrosis [5]. Nephrotoxicity is frequently caused by a variety of drugs and chemicals, or environmental pollutants. Drugs cause approximately twenty five percent of nephrotoxicity, which can increase up to 66% in elderly people [6].

**Figure 1:**
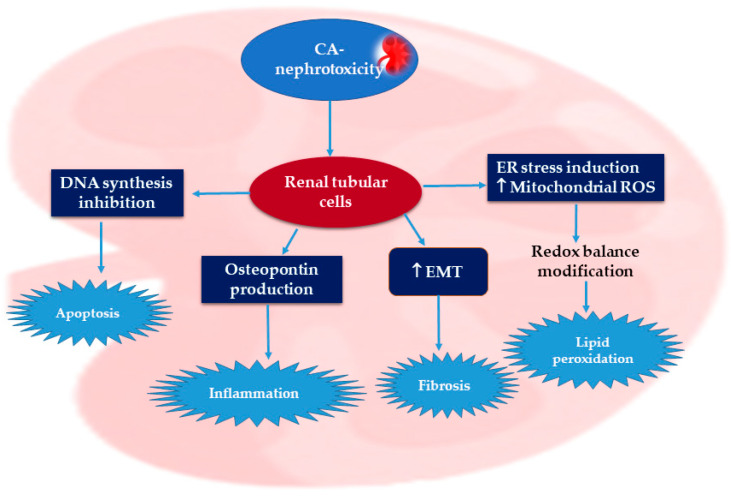
Possible mechanisms of cyclosporine A nephrotoxic effects [13,14,15].

Cyclosporine A (CA), a cyclic peptide consisting of eleven amino acids, is purified from *Topocladium inflatum* fungus. CA is a potent immuno-suppressive agent that is commonly utilized to prohibit transplanted-organ rejection [7]. In solid-organ transplantation, CA significantly improves long-term survival rates [8]. Moreover, CA is utilized to manage varied autoimmune disorders, such as rheumatoid arthritis, psoriasis, and nephritic syndrome, as well as dermatological disorders [9]. However, CA has a narrow therapeutic index and its metabolism is performed by hepatic cytochrome (CYP450 3A 4/5) [10]. Nephrotoxicity is one of the serious adverse effects that limit the therapeutic uses of CA. Several reports have discussed the mechanisms by which CA induces nephrotoxicity [11,12]. The CA-nephrotoxicity-involved mechanism has not yet been completely elucidated. In 2017, a report by Lai et al. indicated that CA mediated renal damage through many mechanisms, involving the generation of inflammation, oxidative stress (OS), autophagy, and apoptosis [13] (Figure 1).

**Figure 2:**
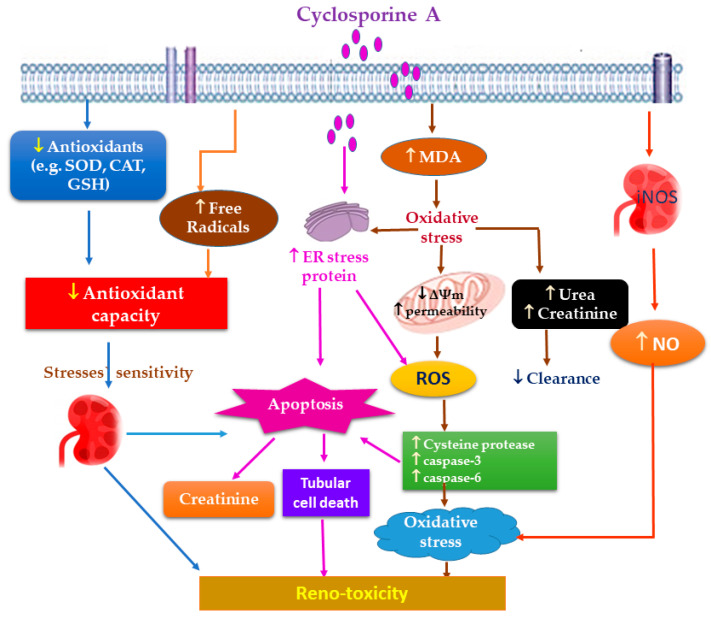
Proposed mechanisms of CA oxidative stress-induced reno-toxicity.

Numerous evidence reveals that ROS overproduction and OS have a definitive role in CA renal pathogenesis [13,14,15]. Briefly, CA promotes endoplasmic reticulum stress and increases the production of mitochondrial ROS (reactive oxygen species), resulting in redox balance alteration, which causes lipid peroxidation (Figure 2) [11].

It has been reported that diverse signaling pathways take part in CA-nephrotoxicity pathogenesis, such as ERK, p38, and JNK, whereas NF-κB represents a CA-target molecule. Moreover, Nrf2 regulates the cellular oxidative stress induced by CA, while renal fibrosis produced by CA is attributed to TGF-β1 (transforming growth-factor-b1) [11,16]. Generally, CA weakens endothelium-based relaxation and prohibits the synthesis of nitric oxide in the renal artery. The nephrotoxic effect of CA is exerted by targeting the epithelial cells of renal tubules and stimulates the EMT (epithelial–mesenchymal transition) in these cells, leading to inflammation-mediated fibrosis and finally kidney failure [17,18]. Moreover, it suppresses DNA synthesis and induces apoptosis in these cells [19]. Shi et al. stated that CA stimulates the production of vasoconstriction factors, such as ROS, RNS (reactive nitrogen species), TGF-β1, NO, angiotensin II, leukotriene, and thromboxane A2 [20]. Moreover, Wirestam et al. reported that CA can indirectly damage renal tubular cells via stimulating osteopontin production, which leads to injuring the renal cells [21]. Therefore, the strategy used to date for overcoming CA is either reducing its dosage or using a combination of CA and another drug. It is noteworthy that various natural metabolites are reported to have the capacity to ameliorate CA-mediated renal toxicity, such as phenolics, polysaccharides, and terpenoids. Thus, the rational use of natural products could assist in minimizing the toxicity of this drug. The current review mainly focuses on natural products that are capable of reducing CA-induced nephrotoxicity. This review introduces a positive perception of the role of natural products in the amelioration of CA-induced nephrotoxicity, thereby amending the treatment strategies for patients who receive CA and their possible implications for natural supplements or drug combinations. The literature was searched using different databases and publishers: Scopus, PubMed, Springer, Google-Scholar, MDPI, Elsevier, Wiley, Bentham, and JACS. The keywords utilized for the search included natural product + cyclosporine A + nephrotoxicity; reno-protective + natural products + cyclosporine A-induced nephrotoxicity; and natural product + nephro-protection + cyclosporine A. No time limit for the publication date was set. A total of 108 articles are reviewed in this study. All the reported studies evaluating the effectiveness of potential natural reno-protective agents on CA-caused nephrotoxicity are summarized.

## 2. Phytoconstituents Prevent CA-Induced Nephrotoxicity

**Figure 3:**
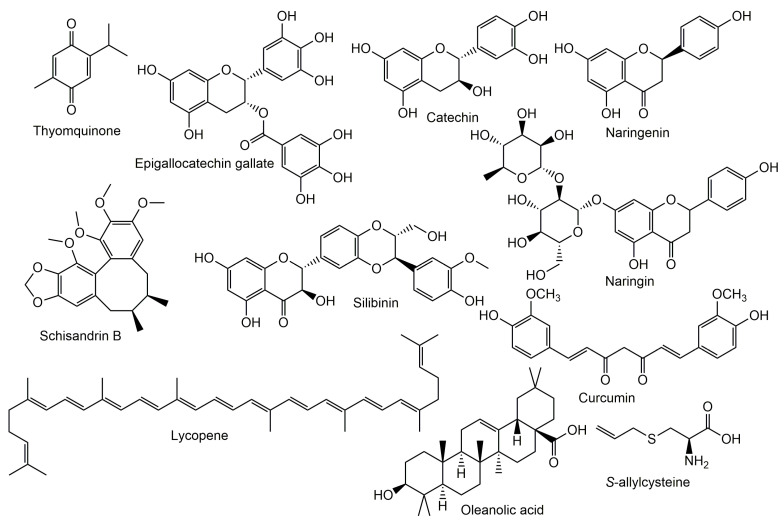
Chemical structures of natural metabolites tested for reno-protective potential.

Different classes of phytochemicals were proved to improve the nephrotoxicity accompanying the use of cyclosporins (Figure 3).

### 2.1. Phenolics and Polyphenols

#### 2.1.1. Catechin

Catechin is a flavanol widely available in many **foods** and herbal products, especially in green tea extract (Table 1). Recent studies revealed that catechin has a dose-dependent nephro-protective effect on CA-induced nephrotoxicity [22]. The administration of CA in a dose of 20 mg/kg/day for twenty-one days markedly affected renal function, as indicated by the increase in renal creatinine and urea levels in rats. Catechin (100 mg/kg/day) co-administered with CA significantly ameliorated the nephrotoxic effect of CA as indicated by enhanced renal functions. On the other hand, lower doses of catechin (50 mg/kg/day) presented a milder protective effect than higher doses of the compound [22]. The prophylactic mechanism of catechin on CA-induced renal toxicity may be due to its antioxidant effect. Oxidative parameters, such as increased lipid peroxidation, decreased glutathione and superoxide dismutase levels, and increased catalase levels, were detected in rat kidney homogenate upon a chronic administration of CA. The co-administration of catechin (50 and 100 mg/Kg/day) with CA markedly enhanced all the previously mentioned oxidative stress parameters in a dose-dependent manner [22].

#### 2.1.2. Epigallocatechin Gallate (EGCG)

EGCG is the most abundant catechin derivative in green tea with marked antioxidant and anti-inflammatory activities. It also has a prophylactic effect on neurodegenerative diseases and diabetes [38]. Similar to catechin, EGCG provides a significant protective effect on CA-induced nephrotoxicity mediated by its antioxidative effect [39]. Despite its biological activities, it has a poor pharmacokinetic property upon oral administration due to its unstable alkaline PH, poor intestinal permeability, and extensive pre-systemic metabolism. Italia et al. compared the effect of an EGCG nanoparticulate formulation on CA-induced nephrotoxicity in rats, in both orally and intraperitoneally administered compounds. I.P. (intra-peritoneal)-administered pure EGCG presented a significant protective effect, while the activity was diminished through the oral administration of the compound (Table 2). On the other hand, the oral administration of an EGCG nano-formulation presented a nephro-protective effect similar to that of an I.P.-administered drug due to its improved pharmacokinetic properties [38].

#### 2.1.3. Naringin

Naringin is a flavanone glycoside that mainly occurs in citrus fruits (Rutaceae), especially grapefruit. It has been reported that naringin is an effective reno-protective agent against CA-induced nephrotoxicity. Naringin can significantly decrease free-radical levels, including OH and lipid peroxidation, and increase antioxidant enzyme (e.g., SOD, GPx, and catalase) activity in renal tissues. Moreover, it can restore non-enzymatic antioxidants (GSH and vitamins C, E, and A). Additionally, it ameliorates the degeneration damage to kidneys induced by CA. Furthermore, the expression of HO-1 is maintained during naringin treatment, which may be the major reason for reno-protection [29].

#### 2.1.4. Silibinin

Silibinin is one of the major flavonolignans in the hepatoprotective drug silymarin, with reported antioxidant activity and an inhibition of rat microsome lipid peroxidation. The effect of the administration of silibinin with CA on malondialdehyde levels in blood and kidney homogenate, in addition to the level of cytochrome p450, an enzyme that metabolizes CA into inactive metabolites, in microsomal liver suspension were estimated. MDA and creatinine levels were returned to normal by the co-administration of silibinin with CA. Interestingly, silibinin administration did not affect the glomerular filtration rate, but markedly increased cytochrome p450 levels compared to the CA group, suggesting its effect on cyclosporine biotransformation in the liver [47].

#### 2.1.5. Ellagic Acid

Ellagic acid is a known phenolic constituent of many fruits and nuts, such as raspberries, strawberries, grapes, and walnuts [51]. It has been reported to present anti-oxidant, anticancer, anti-inflammatory, and antimutagenic effects through its potent free-radical scavenging activity. The subcutaneous administration of ellagic acid (10 mg/kg) revealed a marked protective effect on liver and kidney functions compared to the CA group. Ellagic acid normalized MDA, catalase, and glutathione levels when co-administered with CA. These biochemical results were confirmed further through histopathological investigations [46]. This activity seemed to be dependent on the antioxidant activities of ellagic acid.

#### 2.1.6. Caffeic Acid Phenethyl Ester

Caffeic acid phenethyl ester (CAPE) is a natural phenolic compound formed by the esterification of caffeic acid with phenethyl alcohol. It represents a major component of propolis obtained from honeybees. CA-induced nephrotoxicity produced lipid peroxidation, MPO, SOD, and CAT activities in renal tissue. CAPE prevented an increase in MDA, but increased CAT and did not affect MPO and SOD. Therefore, CAPE may be an efficient agent to protect the kidneys from CA-induced damage via the inhibition of lipid peroxidation [36].

#### 2.1.7. Resveratrol

Resveratrol is a polyphenolic compound that represents a major constituent in red grapes (*Vitis vinifera*, Vitaceae) [52]. It is known for its anti-oxidant, anti-inflammatory, anti-glycation, hepatoprotective, and anti-cancer properties. Moreover, it can improve hyperglycemia, hyperlipidemia, and diabetic complications. A study conducted by Chander et al. revealed that at doses of 5 and 10 mg/kg, resveratrol was able to improve renal dysfunction and renal and tissue nitric oxide levels, as well as renal oxidative stress. This protective effect was proved to be NO-dependent [37].

#### 2.1.8. Provinol

Provinol is a mixture of polyphenolic compounds extracted from French red wine, involving (in mg/g of dry powder) 480 proanthocyanidins, 370 polymeric tannins, 61 total anthocyanins, 19 free anthocyanins, 38 catechins, 18 hydroxycinnamic acids, and 14 flavonols [53]. Provinol was observed to protect against CA-induced renal toxicity due to the antioxidant activity of its phenolic content [49,54]. However, the anti-apoptotic effect of phenolics on renal cells was clarified further in rats treated with CA, provinol, and a combination of both drugs for 21 days. CA markedly increased systolic blood pressure, decreased body weight, and increased serum creatinine and total protein levels. The administration of provinol with CA markedly protected the rats from nephrotoxicity, as indicated by the enhanced biochemical parameters. In addition, the CA group revealed alterations in and the translocation of Bax and cytochrome c levels from cytoplasm to mitochondria that activated the caspase-mediated apoptotic pathway. Provinol markedly inhibited this apoptotic cascade, which was concluded to be the reason for its nephro-protective effect [50]. Provinol could ameliorate a reduction in body weight and increased systolic blood pressure induced by rat treatment with CA. It also exerted a reduction in oxidative stress and iNOS expression via the NF-kB pathway [53].

#### 2.1.9. Curcumin

Curcumin is a diarylheptanoid that represents a major active compound in the rhizomes of *Curcuma longa* (Zingiberaceae). It is traditionally recommended in the treatment of biliary and hepatic disorders and rheumatism. Previous biological studies proved its powerful antioxidant, anti-inflammatory, and antiviral effects. Curcumin proved to present a significant protective effect against CA-induced nephrotoxicity in rats based on its ability to modify all histological changes and antioxidant effects via GST immuno-expression, decreasing TBARS and increasing levels of antioxidant enzymes (GSH, SOD, and CAT) [33,45]. Moreover, it was able to decrease serum creatinine levels and BUN, and improve creatinine clearance [44]. In another study, the treatment of HK-2 human renal cells with CA and different doses of curcumin [44] caused a dose-dependent reduction in ROS and MDA levels, in addition to increasing SOD, GSH-Px, and CAT levels. Moreover, it increased Bcl-2 and decreased Bax protein in HK-2 cells. Moreover, in vitro and in silico studies proved the ability of curcumin to ameliorate genotoxicity as well as DNA damage produced by the long-term use of CA [55]. The mechanism of this effect is based on the ability of curcumin to indirectly induce the expression of different anti-oxidant enzymes. Additionally, curcumin activated Nrf2-Keap1 that is responsible for the free-radical eradication from tissues through the expression of detoxifying enzymes [55].

### 2.2. Lignan Derivatives

Schisandrin B (ScB), a lignan derivative, was separated from *Schisandra chinensis*. This plant displayed beneficial values in treating hepatitis and regulating renal function [13]. Its extract had protective potential against nephrotoxicity caused by CA in rats [25]. ScB was reported to have a protective effect against cisplatin, gentamicin, and mercury-mediated nephrotoxicity [56,57,58] in rats. In a study on HK-2, the cyto-protective influence of ScB towards the CA nephrotoxic effect by assessing different parameters, such as LDH, GSH, ROS, and ΔΨm (mitochondrial membrane potential), as well as apoptosis and autophagy, was investigated [13]. It was revealed that the pre-incubation of HK-2 cells with ScB (2.5–10.0 μM) alleviated the cytotoxicity caused CA because of OS, as it reduced ROS and LDH levels and increased ΔΨm and GSH. Furthermore, it stimulated the translocation of Nrf2 into the nucleus and downstream HO-1, NQO1, and GCLM gene expressions, as well as reducing the apoptosis rate and recovering the blocked auto-phagic flux induced by CA. Therefore, ScB has a remarkable role in prohibiting CA-provoked OS, autophagy, and apoptosis by promoting cell survival through ROS scavenging [13]. Another study conducted by Zhu et al. in 2012 to assess the effect of ScB on CA produced renal toxicity both in vivo and in vitro. ScB (20 mg/kg/day, gavage followed by CA 30 mg/kg/day, SC for 28 days) significantly repressed the increase in serum creatinine and BUN levels, and improved the kidney structure alteration caused by CA in mice. ScB also reversed the CA negative effects, as indicated by decreasing renal MDA levels and increasing GSH levels. In vitro, Sch B (2.5, 5, and 10 µM) prominently increased HK-2 cell viability and decreased apoptosis and the release of LDH provoked by CA (10 µM), as well as increased ATP and GSH intracellular levels and attenuated ROS generation induced by CA. It is noteworthy that the reduction in OS and cell death rates was proposed to be the reason for ScB’s protective effect [41].

### 2.3. Carotenoids

LYC (lycopene), a carotenoid, is accountable for the pink-to-red colors of grapefruits, tomatoes, and other foods. It presents a protective influence against various chronic disorders, such as skin, prostate, and lung cancers, as well as degenerative and cardiovascular diseases [59]. LYC is known to have a potent ROS-quenching power and protects DNA, proteins, and lipids against oxidation in vivo [60,61]. It has a protective effect on gentamicin-induced renal damage in rats [62]. Gado et al. conducted a study that revealed the protective effect of LYC (40 mg/kg/day/p.o. for 5 days before and 10 days concomitant with CA) against nephrotoxicity induced by CA. The results show that LYC significantly reduces creatinine and urea serum levels and restores GSH content, as well as prohibits MDA elevation and increases SOD and GSH-Px activities. A histological investigation revealed the amelioration of nephritis and tubular necrosis in comparison with the CA group. LyC alleviated kidney impairment caused by the CA oxidative stress mechanism due to its antioxidant potential [26]. In another investigation, performed in 2007, Ateșșahin et al. evaluated the renal protective action of LYC (10 mg/kg/day for 21 days) in the renal damage and oxidative stress caused by CA in rats, as indicated by the increase in plasma urea and creatinine levels, as well as increased TBARS and GSH and decreased CAT and GSH-Px activities. Moreover, degeneration, tubular necrosis, dilatation, formation of luminal cast, thickened basement membranes, and inter-tubular fibrosis were observed in CA-intoxicated rats. LYC treatment ameliorated the CA reno-toxic effect via decreased plasma urea and creatinine concentrations and elevated TBARs levels while it increased GSH-Px and CAT activity. Moreover, it restored the pathological alteration produced by CA in the kidneys [42].

### 2.4. Organo-Sulfur Derivatives

S-allylcysteine (SAC), an organo-sulfur constituent of aged *Allium sativum*, exhibits antioxidant, anti-cancer, neuro-trophic, hepato-, and cardio-protective properties via its antioxidant potential [63]. It was stated that SAC is able to scavenge H_2_O_2_ and O_2_, thus prohibiting H_2_O_2_-induced endothelial cell damage, LPO, and LDL low-density lipoprotein oxidation [27]. In 2008, Magendiramani et al. investigated the protective potential of an SAC (100 mg/kg/day, I.P.) co-injection on AC (25 mg/kg/day, I.P.)-induced nephrotoxicity in a rat. The results indicate a marked elevation in uric acid, urea, and creatinine serum levels and LPO together with abnormal antioxidant (non-enzymatic; vit. E and C and GSH, and enzymatic CAT, GPx, SOD, and GR) levels in the CA group in comparison to the control group. Their results reveal that SAC significantly attenuates peroxidative levels and boosts the antioxidant status along with reducing iNOS, MMP-2, and NF-kB elevated levels because of CA. Moreover, it decreases the observed increase in uric acid, urea, and creatinine levels, as well as inflammation and renal injury in CA-treated rats [27].

### 2.5. Terpenoids

Thymoquinone (TQ), a component of *Nigella sativa* oil, has anti-hyperglycemic, nephro- and hepato-protective, anti-inflammatory, hypolipidemic, and anti-neoplastic activities. It prohibits CYP3A that is accountable for metabolizing most drugs [64]. Alrashedia et al., in a study performed in 2018, revealed that TQ (orally, 10 mg/kg, 7 days) reduced the bioavailability of oral CA (10 mg/kg, 5 days) and had no effect on the bioavailability of IP-administered CA (10 mg/kg, 5 days, 1 hr after TQ) in rats because of the induction of intestinal first-pass metabolism by TQ, which in turn reduced its blood concentration, resulting in a marked reduction in its nephrotoxicity. On the other hand, TQ significantly attenuated the CA-produced reno-toxic effect, including a reduction in serum creatinine and cystatin C levels and improving kidney tubular and glomerular renal structures [23]. The TQ protective effect may refer to its antioxidant capacity. Hussein et al. also assessed the reno-protective effect of TQ (10 mg/kg b. wt./orally/day for 7 days) against CA (oral dose 25 mg/kg/b.wt./day)-mediated renal toxicity in rats. It was found to normalize increased levels of L-MDA in renal tissues and creatinine and urea in serum as well as the decreased catalase activity and GSH level. Moreover, it markedly upregulated Bcl-2 and downregulated PAI-1, NF-κB, p53, and caspase-3 gene expressions levels. Furthermore, TQ remarkably improved the renal damage and OS alterations via its anti-apoptosis, anti-inflammatory, and antioxidative properties [65].

Oleanolic acid is a triterpene pentacyclic carboxylic acid separated from *Olea europaea* that possesses hepato-protective, anticancer, and anti-inflammatory potential [24]. It presents a generalized protective effect against chronic cyclosporine nephropathy through Nrf2/HO-1 pathway upregulation resulting in increased levels of NQO-1, HO-1, GSH, SOD, GCL, and S-transferase via affecting the ARE gene, thereby decreasing apoptosis and degradation. This suggests that oleanolic acid could be a potential therapeutic agent for treating A-induced nephrotoxicity involving ARE/Nrf2/HO-1 [24].

### 2.6. Polysaccharides

Sulfated polysaccharides are a type of metabolite having ester-linked sulfate groups in their backbone. They are commonly reported in seaweeds and have various therapeutic applications [66,67]. They are glycosaminoglycans that can positively counteract glomerular disorders. It has been stated that they possessed an antioxidant potential and have a remarkable mitochondrial influence via their enhanced antioxidant state, decreased accumulation of ROS, improved mitochondrial membrane potential and ATP status, and prohibited release of cytochromes [68,69,70].

Genus *Sargassum* seaweed is a rich pool for their bioactivities with remarkable biomedical and pharmaceutical uses [71]. *Sargassum wightii*-sulphated polysaccharides (SWSPs) possess hypolipidemic effects, thus reducing the risk of glomerular dysfunction-associated hyperlipidemia [72].

A study conducted by [69] revealed that SWSPs (5 mg/kg/b.w., SC., for 21 days) possess a protective potential against CA-mediated nephrotoxicity (orally 25 mg/kg/b.w.), as indicated by improved body weight, normalized lysosomal enzymes and creatinine clearance, and attenuated morphological alterations in renal tissues caused by CA in rats [69]. Another study conducted by the same authors demonstrated that SWSPs modulate CA-induced mitochondrial dysfunction and tubular injuries via its powerful antioxidant effect, notably prohibiting mitochondrial oxidative stress through scavenging free radicals, boosting GPx and SOD, and improving the GSH levels. It also suppresses LPO and mitochondrial swelling [68].

## 3. Herbal Extracts Prevent CA-Induced Nephrotoxicity

Natural products, especially herbal extracts, have a marked role in folk medicine as protecting the kidneys due to their significant antioxidant and anti-inflammatory effects. Recently, several in vitro and in vivo models were implemented to discover the kidney-protective components of plants [1].

### 3.1. Zingiber officinale

*Zingiber officinale*, commonly named ginger, is a perennial plant that belongs to the Zingiberaceae family. The rhizome of the plant has a wide range of medical applications in the treatment of motion sickness, inflammation, and cancer [73]. The activity of ginger rhizomes could be attributed to their polyphenol contents that may be responsible for the antidiabetic, cardio-protective, and hepato-protective activities of the plant [74,75]. In addition, the polyphenol-rich extract (prepared using 80% acetone) could attenuate CA-induced disturbances in kidney function. The prepared extract reversed all alterations produced in the kidneys by CA through a significant improvement in the plasma and urine levels of creatinine, urea, Na+ and K+ electrolyte balance, as well as creatinine clearance. Moreover, it improved feeding patterns, relative kidney weight, and oxidative stress (GSH and SOD). These improvements were also confirmed by a histopathological study [76].

### 3.2. Phoenix dactylifera

The fruits of *Phoenix dactylifera* or date palm are widely used as food in many Middle-Eastern countries. Date pits, a byproduct of date palm, were found to be rich in polyphenolic compounds and exert antioxidant, antibacterial, and chemoprotective activities [77]. The protective effect of date pit aqueous extract (DPE) on CA-induced nephrotoxicity was studied. DPE enhanced kidney function after CA administration and increased glutathione levels. A marked decrease in LPO and increase in CAT levels were observed. These results were further confirmed through histopathological investigations of kidney tissues. It was proposed that DPE restored kidney functions in CA-induced nephrotoxicity in rats through antioxidant mechanisms [77].

### 3.3. Spinacea oleracea

*Spinacea oleracea* (spinach) is a green, leafy plant used as a food in many countries all over the world. It possesses antioxidant properties, and inhibits lipid peroxidation and hepatoprotective effects in CCl_4_-induced liver toxicity [78]. *N*-Hexane extract from spinach leaves was administered with CA for 14 days, and the results of the histopathological investigation of kidney tissues were compared to both CA and spinach-only groups. CA-treated groups presented marked kidney toxicity through vacuolation, necrosis, and loss of brush border in tubular cells. The co-administration of spinach with CA revealed a significant amelioration of all these histopathological lesions, suggesting that spinach hexane extract has a protective effect on CA-induced nephrotoxicity [78].

### 3.4. Ginseng

Ginseng is widely used in many countries due to its well-known biological activities. The antioxidant activity of ginseng constituents has been discussed and documented in many reports. The protective effect of Korean red ginseng extract (KRG) on CA-induced nephrotoxicity in a mouse model was investigated by measuring renal function, inflammatory mediators, and tubular fibrosis and apoptosis [79]. In addition, the effect of KRG on CA-treated proximal tubular cells (HK-2) was investigated in vitro. 8-Hydroxy-2′-deoxyguanosine (8-OhdG) in urine and tissues was used as a measure of oxidative stress. KRG treatment decreased creatinine levels and proinflammatory mediators, such as NO synthase and cytokines. Induced cellular apoptosis was also decreased by KRG treatment. Moreover, 8-OhdG levels were markedly decreased in urine and tissue samples following KRG administration (Table 3). It was concluded that KRG exerts its nephro-protective effect through antioxidant activity and the prevention of apoptosis [79].

### 3.5. Grape and Garlic

Black grapes and garlic are well-known antioxidant foods due to their allicin, alline, and resveratrol contents, respectively. The protective effect of dried black grapes and garlic aqueous extracts on CA-induced nephrotoxicity was investigated in rats by Durak et al. [81]. The grapes and garlic extract were given three days before CA administration for 10 days, and oxidative stress parameters in addition to histopathological investigations were performed. The administration of both plants reduced MDA levels in kidney tissues through the prevention of oxidative stress. Moreover, in a different study, the ingestion of 25 g/kg of dried black grapes with CA by rats significantly decreased MDA levels in the kidney tissues of rats. However, no significant difference was observed in SOD and catalase levels [91]. In the same context, Hussein et al. studied the protective effect of grape seed proanthocyanidin-rich extract (GSPE) on CA-induced nephrotoxicity. GPSE was standardized to contain 66.7 mg/g of total phenolics with an oligomeric proanthocyanidin ratio of 95%. GSPE extracts (200 mg/kg) was administered 7 days before and 21 during CA administration in rats [92]. GSPE treatment decreased serum creatinine, urea, and tissue MDA levels, and reduced glutathione levels. In addition, GSPE treatment retained Bcl-2, NF-κB, caspase-3, and P53 to their normal levels. Thus, grape seed extracts exerted their effect through antioxidant and anti-inflammatory properties, and the inhibition of apoptosis. Similar to other studies, GPSE ameliorated impaired kidney function upon co-administration with CA through its antioxidant properties and inhibition of apoptosis. In addition, GPSE did not affect CA plasma levels after administration [93].

Aged garlic extract (AGE) is an odorless material produced by the extraction of garlic for a long period of time (20 months) [93]. AGE was proved to be the most potent antioxidant among all garlic preparations. AGE in 0.25, 0.5, 1, and 2 g/kg was administered 3 days prior to CA treatment, followed by 10 days of co-administration. AGE in doses of 0.5–2 g/kg decreased renal creatinine and increased creatinine clearance, and ameliorated histopathological changes, such as vacuolation and tubular necrosis [94].

### 3.6. Green Tea

Tea is the most commonly consumed beverage worldwide, with a known abundance of polyphenol contents. The most abundant compounds in green tea are EGCG and catechin, which are known for their well-reported antioxidant activities. Green tea extract (GTE) with a concentration of 3% W/V was orally administered 21 days before CA and administered for 21 days with CA followed by 21 days alone. GTE was found to alleviate all kidney toxicity parameters, such as increasing GSH and catalase levels and decreasing MDA, creatinine, and urea levels. In addition, it ameliorated the lipid profile and serum glucose, LDH, and GGT levels affected by CA administration [82]. In another study by Mohamadin et al. [83], a 0.5, 1, and 1.5 % W/V solution prepared from instant lyophilized green tea powder was consumed by rats in the experiment 4 days before CA and concurrent with it for 21 days. In addition to the usual enhancement of kidney function and oxidative parameters, GTE inhibited the activity of lysosomal enzymes NAG, β-GU, and AP [83].

### 3.7. Ipomoea batatas

An aqueous leaf extract of *Ipomoea batatas* was orally administered in 200 and 400 mg/kg in rats concurrently with CA. It alleviated the CA-induced increase in serum inflammatory cytokines and kidney functions. Moreover, it retained the normal ionic sodium and potassium levels compared to the CA group, in addition to enhancing the impaired histopathological status of kidney tissues by CA [88].

### 3.8. Schisandra chinensis

In China, patients treated with CA are advised to consume pharmaceutical preparations containing *Schisandra chinensis* for protection from its side effects [95]. The plant is reported to contain several triterpenoids, such as schisandrol A, schisantherin A, schizandrin A, and schizandrin B. The administration of *Schisandara* extract (SCE) alleviated hepatorenal injuries induced by CA through the activation of the Nrf2 pathway and the inhibition of apoptosis [90].

### 3.9. Nigella sativa

Black seed (*Nigella sativa*) is widely used for culinary and medicinal purposes. Several reports confirmed the protective effects of *Nigella sativa* extract and its main constituent, thymoquinone, on cisplatin-induced nephrotoxicity [96,97]. *Nigella sativa* oil (NSO) in a dose of 2 mL/kg was co-administered with CA to rats, and their kidney function and oxidative stress parameters were investigated. NSO significantly improved renal functions as deduced from lowering serum creatinine and urea levels. In addition, oxidative parameters were markedly improved, such as tissue MDA, CAT, glutathione, and SOD levels. Moreover, the biochemical effects of NSO were confirmed further through the histopathological improvement of kidney tissues. Therefore, NSO ameliorated CA-induced nephrotoxicity through its possible antioxidant effect.

### 3.10. Cordyceps sinensis

*C. sinensis* is a plant widely used in Chinese folk medicine as a kidney tonic. The effect of the plant’s administration on the protection of kidneys from the toxic effects of CA in patients with transplantations was studied. The concurrent administration of *C. sinensis* and CA resulted in significantly reduced nephrotoxicity compared to the CA group of patients, as indicated by decreased serum creatinine, urea, and NAG levels. Moreover, CA plasma levels were the same in both groups [86].

### 3.11. Doum, Carob, and Fennel

Doum, carob, and fennel are edible plants widely used in Egypt for their culinary and medicinal properties. Fennel (*Foeniculum vulgare*) is widely cultivated in the Mediterranean region and used for its aroma and flavor in salad and many dishes, and for its antioxidant, antispasmodic, and antiflatulence properties in folk medicine [98]. Carob (*Ceratonia siliqua*), which belongs to the Fabaceae family, is widely used in the Mediterranean region as a beverage and food due to its carbohydrate, fiber, and phenolic contents [99]. Doum (*Hyphaene thebaica*) is a desert palm native to Egypt, Africa, and India. Its fruit pulp is widely used due to its minerals, phenolics, and linoleic acid content [100]. A recent study focused on its possible antihypertensive effects [101]. After the initial injection of rats with CA for 7 days, hey were allowed to consume food containing the three plants. The use of fennel, doum, and carob decreased serum creatinine levels; urinary levels of β2 microglobulin; and serum levels of ammonia, TGF-β1, and TNF-α; and decreased creatinine clearance. Furthermore, a histopathological assessment confirmed the protective effects of these plants through their possible anti-inflammatory effects [87].

## 4. Miscellaneous Natural Products

### 4.1. Propolis 

Propolis is a bee product rich in a variety of natural constituents, mainly phenolics. Propolis gained popularity as an antioxidant and food additive that is used for treating several diseases. The 60% hydroalcoholic extract of propolis was studied for its nephro-protective effect against CA-induced kidney dysfunction in rats. Propolis extract was administered with CA in a dose of 100 mg/kg orally. It was found that serum cortisol, AST, ALT, and urea levels were markedly decreased upon propolis administration. Moreover, propolis decreased kidney and liver MDA levels, and increased catalase and reduced GSH levels [84].

### 4.2. Spirulina

Spirulina (*Arthrospira platensis*) is a filamentous blue–green microalgae that acquired its name from its spiral-shaped filaments. It contains carbohydrates, proteins, vitamin B, minerals, and carotenoids, such as beta carotene. It has antioxidant, anti-inflammatory, and nephro- and radioprotective activities. Spirulina at 1 g/kg was administered 15 days before irradiation or 5 days before and 10 days with CA. Gamma radiation and CA induced a marked elevation in serum creatinine, urea, lipids, and glucose levels, which was reversed by spirulina intake. In addition, spirulina increased kidney SOD and decreased MDA and nitrile levels. Biochemical parameters were confirmed further through histopathological studies. Moreover, kidney caspase-3 levels in the CA-treated group were significantly decreased by using spirulina [85].

## 5. Diet Prevents CA-Induced Nephrotoxicity

Less research has been conducted to assess the effect of dietary regimen on CA-induced reno-toxicity. We presented the results of these studies in the present paper.

Fish oil-derived omega-3 fatty acids have a protective potential against various metabolic disorders and diseases, such as MetS, cancer, neuro-degenerative and autoimmune disorders, diabetes, and CVD [102]. They play a significant role in anti-inflammatory processes and improve the antioxidant defense system [103]. Priyamvada et al. reported that dietary fish oil (DFO) alleviated gentamicin-produced oxidative damage and metabolic alterations because of its intrinsic antioxidant/biochemical properties [104,105]. In 2014, Hussein et al. demonstrated that CA remarkably increased the renal function tests, serum glucose, lipid profiles, haptoglobin, and serum (GGT and LDH) enzymes with a considerable lowering of serum albumin, electrolytes, and total protein. Co-treatment with DFO and CA remarkably reduced these parameters as compared with the CA-received group. Moreover, CA induced a considerable increase in MDA, along with a noticeable reduction in enzymatic and non-enzymatic antioxidants, TOC, and NO levels in the rat kidneys. Meanwhile, DFO improved renal function through a significant increase in the antioxidant status and decrease in peroxidative levels. These results reveal the usefulness and reno-protective capacity of DFO as a rich source of antioxidants in modulating CA-induced nephrotoxicity [40]. Moreover, it was reported that the antioxidant nutrients, such as vitamins C and E, ameliorate the toxic effects produced CA in kidneys, whereas vitamin E prohibits ROS and TX synthesis as well as lipid peroxidation caused by CA. Furthermore, they can improve renal function and CA-produced histological damage [106]. A study by Klawitter et al. in 2012 demonstrated that low-salt-diet-fed rats are more sensitive to CA (10 mg/kg/day CA for 28 days on low-salt diet) renal injuries than normal-salt-diet-fed rats (10 mg/kg/day CA for 28 days on low-salt diet). Their results show that micro- and macro-vesicular tubular epithelial vacuolizations and a reduced energy charge are more prominent in low-salt-fed rats. CA increased phospho-JAK2 and -STAT3 levels and reduced p65 and phospho-IKKγ proteins, leading to NF-κB signaling activation. Moreover, reduced lactate transport regulator CD147 and phospho-AKT expression were noted after the exposure of low-salt-fed rats to CA, revealing a decrease in glycolysis. Collectively, AKT, CD147, and JAK/STAT signaling displayed a remarkable role in CA reno-toxicity [107].

A protein-rich diet’s potential for several drugs producing renal toxicity was investigated. Protein feeding was reported to increase GFR and RPF levels in rats [108]. Therefore, it may counteract the vasoconstriction produced by CA and reduce its nephrotoxicity. A study performed by Pons et al. in 2003 demonstrated the protective effect of a casein-rich diet against in proximal tubule damage induced by CA. In CA (25 mg/kg/24h, I.P. for 7 days)-challenge rats, there were no significant differences in caloric consumption, bodyweight, urine output, and water intake among standard Rat Chow and high-protein fed (casein-rich diet for 2 weeks before CA) animals. However, β-GAL and NAG urine excretion and renal post-necrotic cellular regeneration were remarkably lower for the high-protein diet CA-treated rats than in those fed with standard Rat Chow, and no gold particle was observed over proximal tubule lysosomes in rich-protein-diet-fed rats [109]. Venkateswarlu et al. evaluated the LOBUN probiotic formulation’s (500 mg/kg/b.w. for twice or thrice a day from the 15th to 28th day) nephro-protective effect on CA (20 mg/kg SC, 15 days)-induced renal impairment in Wistar rats. In this study, CA-induced renal toxicity was indicated by increased BUN, serum creatinine, uric acid, and total protein levels, as well as urine potassium, proteins, and sodium. LOBUN (500 mg/kg b.w. thrice a day) provided appreciable reno-protection and alleviated the CKD symptoms against CA that was evidenced by biochemical and histological findings [80].

Olive oil is regarded as a superfood with numerous health benefits that are attributed to its unique contents, including high percent of MUFA (monounsaturated fatty acids) as well as other bioactive constituents. Its phenolics are reputed to have the potential as anti-inflammatory, antimicrobials, and antioxidants [110]. Elshama et al. investigated the protective potential of VOO (virgin olive oil, 1.25 mL/kg/day, GL) or naringenin (100 mg/kg/day, GL) co-administration on CA (25 mg/kg/day, GL)-induced renal damage in rats. The results reveal that VOO modulates CA-induced ultra-structure and morphologic changes, improves antioxidant status, and decreases urea and creatinine levels to the same extent as naringenin [28].

## 6. Natural Products’ Stability and Adverse Effects

The stability of herbal products as extracts or purified compounds represents an important issue in using natural products to combat ailments. The stability of herbal products includes its ability to preserve its identity, strength, and purity. However, during the process of extraction and preparation of natural products, the active constituents are subjected to oxidation, hydrolysis, microbial attack, and other environmental deterioration effects, which affect its stability [111]. The quality, effectiveness, and shelf life of natural medicines are all impacted by the presence and concentration of bioactive ingredients; therefore, monitoring their presence and concentration is crucial. The factors that may affect the stability of herbal products include: its presence in a complex mixture of different components, drug interaction, or decomposition during storage; physical and chemical stability; and finally the environmental factors. Different techniques could be used to overcome the instability of natural preparations, including nanoparticle coating to enhance shelf life, semisolid preparations based on supercritical carbon dioxide, liquid preparation coated with water-soluble cellulose, derivatives using polymeric plant-derived excipients in drug delivery, micro-encapsulation for active constituents, and adding antioxidants to prevent the oxidation of active compounds. A detailed discussion of the different methods for increasing stability have been previously published [111].

Many people think that pharmaceutical agents are too expensive and have unwanted side-effects; on the other hand, they believe that medicinal herbs must be efficient and safe. However, this prevalent faith that herbs are safe is shown to be faulty. Unfortunately, the contamination by mycotoxins, microbes, pesticides, and even heavy metals, such as arsenic, lead, or mercury, has been reported, especially among Internet-sold herbs [112]. Indeed, several studies reported the hepato-toxic potential of natural products. For example, black cohosh mediated liver injury through mitochondrial damage. EGCG (epigallocatechin gallate), the main phenolic in green tea, was reported to be the most potentially hepatotoxic constituent; in addition, green tea extract’s high doses may cause acute intensive liver injury. Additionally, several potentially dangerous interactions between herbs and drugs have been described [113]. Kava, valerian, St. John’s wort, and ginkgo, which are used for supporting mental health, interact with commonly utilized medications, e.g., the use of St. John’s wort with selective serotonin reuptake inhibitors could result in serotonin syndrome [114]. The CA’s bioavailability was found to be affected by many herbal extracts and traditional drugs that influence CA’s blood concentration. In a case report, St John’s wort and in vivo animal studies, liquorice, ginger, quercetin, and scutellariae radix were shown to decrease CA blood concentration. However, an increased CA concentration was noted with berberine, resveratrol, grapefruit juice, chamomile, or cannabidiol in animal studies [9]. On the other hand, it was stated that the concomitant use of *Serenoa repens* and *Echinacea* with CA should be avoided. Thus, the knowledge of a patient’s usage of natural products before CA administration is crucial to overcome the possible interactions between CA and herbal preparations.

## 7. Conclusions

**Figure 4:**
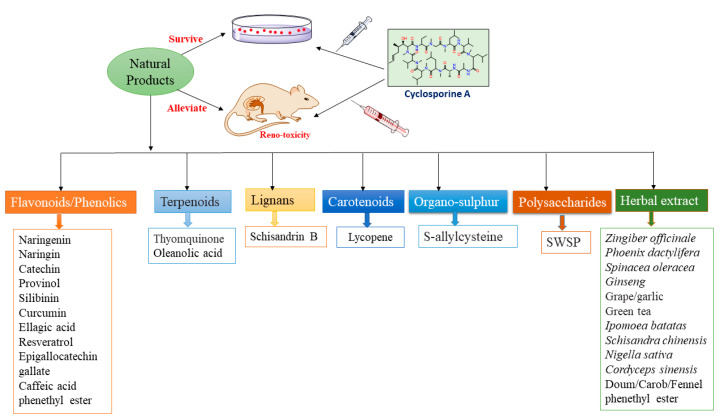
Different classes of natural products protect against CA-induced nephrotoxicity.

Kidneys have an essential role in maintaining homeostasis. Kidney illnesses are serious health concerns that cause an economic burden and worrisome morbidity. They can occur following certain medications’ usage, such as CA that mediates its destructive effect through various cascades. Unfortunately, the pathogenesis of CA-induced reno-toxicity is complicated; its earlier diagnosis is difficult and effective treatment options are lacking. This has encouraged researchers to search for natural metabolites with fewer side effects. It was observed that several natural biomolecules have been reported to reduce or mitigate the severity of CA-induced renal toxicity (Figure 4).

These metabolites appear to have a crucial role in protecting and detoxifying renal tissues against CA-induced damage through their anti-apoptosis, anti-inflammatory, and antioxidative properties. The presented data in this work provided a scientific bases for the rational utilization development and discovery of phytoconstituents for treating practices. In this literature survey, it was observed that the reno-protection of the most-studied plants or their phytoconstituents was explained as related to oxidative stress. Meanwhile, there are other mechanisms of reno-protection that may be responsible for the protective effect on the kidneys, which need further study. Additionally, some studies revealed that dietary regimen has a marked effect on CA-induced reno-toxicity. Most of the reported studies were conducted on animal models. Understanding how natural products act through various signaling pathways will produce a better insight into the potential prevention and treatment of CA-induced renal toxicity. However, further clinical studies are warranted to amend the pharmacokinetic and pharmacodynamic understanding of these metabolites. Additionally, a further evaluation of other classes of natural metabolites reported by various sources is required.

List of abbreviations: 2-DG: 2-Deoxy-D-glucose; 8-iso-PGF2α: 8-epi-prostaglandin F2α; ALP: alkaline phosphatase; AP: acid phosphatase; 8-OHdG: 8-hydroxy-2′-deoxyguanosine; AREs: antioxidant-response elements; b.w.: body weight; BUN: blood urea nitrogen; CA: cyclosporine A; CAT: catalase; c-GT; c-Glutamyl transferase; CVD: cardiovascular disease; CKD: chronic kidney disease; CMC: carboxy methyl cellulose; Cr: creatinine; CYP3A: cytochrome P450, family 3, subfamily A; EGCG: epigallocatechin gallate; EMT: epithelial–mesenchymal transition; GCLM: glutamate-cysteine ligase-modifier subunit; GFR: glomerular filtration rate; GGT: gamma glutamyl transferase; GL: gastric lavage; GSH-Px: glutathione peroxidase; GR: glutathione reductase; GSH: reduced glutathione; GST: glutathione-*S*-transferase; HK-2: human proximal tubular epithelial cell line; HO-1: heme oxygenase-1; HSP-70: heat shock protein-70; I.P.: intra-peritoneal; iNOS: inducible nitric oxide synthase; LDH: lactate dehydrogenase; c-GT: c-Glutamyl transferase; LDL: low-density lipoprotein; LPO: lipid peroxidation; MMP-2: matrix metalloproteinase-2; MetS: metabolic syndrome; MUFAs: monounsaturated fatty acids; NAG: *N*-acetyl-β-d-glucosaminidase; NF-kB: nuclear factor kappa B; NO: nitric oxide; NPs: nanoparticles; NQO1 NAD(P) H: quinone oxidoreductase 1; Nrf2: nuclear factor erythroid 2-related factor 2; OHdG: 8-Hydroxy-2′-deoxyguanosine; OS: oxidative stress; p.o.: perorally; PC: plasma creatinine; PLs: phospholipids; PLGA: poly(lactic-co-glycolic) acid; Px: peroxidase; ROS: reactive oxygen species; RPF: renal plasma flow; SC: subcutaneously; SBP: systolic blood pressure; ScB: Schisandrin B; SWSPs: Sargassum wightii-sulphated polysaccharides; TAO: total antioxidant capacity; TBARS: thiobarbituric acid-reactive substances; TC: total cholesterol; TAGs: triacylglycerols; TP: total protein; TX: thromboxane; TGF-β1: transforming growth factor-β1; TQ: thymoquinone; UA: uric acid; VOO: virgin olive oil; XO: xanthine oxidase; β2MG: β2-microglobulin; β-GAL: β-galactosidase; ΔΨm: mitochondrial transmembrane potential.

## Figures and Tables

**Table 1:** List of natural compounds evaluated for protective effects against CA-induced renal injury.

Compound Name/Class	M.F.	M.W.	Plant/Name (Organ, Family)	Ref.
Terpenoids				
Thyomquinone	C_10_H_12_O_2_	164	*Nigella sativa* (Ranunculaceae)	[23]
Oleanolic acid	C_30_H_48_O_3_	456	*Olea europaea* (Oleaceae)	[24]
Lignans				
Schisandrin B	C_23_H_28_O_6_	400	*Schisandra chinensis* (Schisandraceae)	[25]
Carotenoids				
Lycopene	C_40_H_56_	536	*Psidium guajava* (Myrtaceae) *Solanum lycopersicum* (Solanaceae)	[26]
Organo-sulphur				
S-allylcysteine	C_6_H_11_NO_2_S	161	*Allium sativum* (Amaryllidaceae)	[27]
Flavonoids				
Naringenin	C_15_H_12_O_5_	272	Citrus fruits (Rutaceae)	[28]
Naringin	C_27_H_32_O_14_	580	Citrus fruits (Rutaceae)	[29]
Catechin	C_15_H_14_O_6_	290	*Camellia sinensis* (Theaceae)	[30]
Epigallocatechin gallate	C_22_H_18_O_11_	458	*Camellia sinensis* (Theaceae)	[31]
Silibinin	C_25_H_22_O_10_	482	*Silybum marianum* L. (Asteraceae)	[32]
Phenolics				
Curcumin	C_21_H_20_O_6_	368	*Curcuma longa* (Zingiberaceae)	[33]
Ellagic acid	C_14_H_6_O_8_	302	*Punica granatum* (Punicaceae)	[34,35]
Caffeic acid phenethyl ester	C_17_H_16_O_4_	284	Propolis	[36]
Resveratrol	C_14_H_12_O_3_	228	*Vitis vinifera* (Vitaceae)	[37]

M.F.: molecular formula; M.W.: molecular weight.

**Table 2:** Protective effects of natural compounds on CA-induced renal injury.

Compound	Experimental Model	Intervention (Dose, Route/Duration of Administration)	Studied Parameter	Pharmacological Outcomes/Effects	References
Thymoquinone	Male Wister rats	Four groups Control: vehicle TQ: TQ 10 mg/kg, orally TQ+CA: TQ (10 mg/kg, orally)/1 week then CA (10 mg/kg, I.P.)/5 days with continuing TQ CA: 10 mg/kg, I.P./5 days Treatment duration: 28 days	Serum creatinine Serum cystatin C Blood glucose	Concomitant administration of TQ with CA ↓ serum cystatin and blood glucose levels TQ prevented the major structural changes in both glomerular and tubular components induced by CA	[23]
	White male albino rats	Three groups Control (no drugs) CA: CA 25 mg/kg/day/orally/21 days TQ protected + CA: TQ (10 mg/kg/day, orally) for 7 days before and during CA (25 mg/kg/day/orally) treatment Treatment duration: 21 days	Serum urea and creatinine L-MDA and GSH levels CAT activity NF-kB and PAI-1 expression levels Caspase-3 p53 Bcl-2	TQ ↓ caspase-3 and p53 and ↑ Bcl-2 gene expressions TQ ↓ NF-κB and PAI-1 gene expressions TQ ↓ L-MDA level and ↑ GSH level and CAT activity TQ ↓ urea and creatinine values	[40]
Oleanolic acid (OA)	Five-week-old male ICR mice	Four groups Control: 1 mL olive oil/kg/daily, SC VH + OA: 1 mL olive oil/kg/daily, SC then OA (25 mg/kg/daily I.P. for one week) CA: CA 30 mg/kg/ daily, SC CA + OA: CA (30 mg/kg/ daily, SC) + OA (25 mg/kg/daily I.P.) Treatment duration: 28 days	Renal functional parameters Morphological changes Nrf-2, Keap1, and antioxidant defense system Renal apoptosis	OA ↓ tubulointerstitial fibrosis and inflammation OA ↓ urinary 8-OHdG and 8-iso-PGF2α levels OA ↑ ratio of nuclear/total Nrf2 and enhanced HO-1 expression OA ↓ expression of Bcl-2 and ↑ Bax and cleaved caspase-3	[24]
Schisandrin B (ScB)	HK-2 cell line	Pretreatment with ScB (2.5, 5.0, and 10.0 μM) for 12 h, then CA (10 μM) for 24 h	ROS, Nrf2, Bax, Bcl-2, LC3, Beclin1, and p62	ScB ↓ ROS and LDH levels ScB ↑ ΔΨm and GSH levels ScB activated Nrf2 ScB ameliorated apoptosis induced by CA ScB abrogated autophagy activation stimulated by CA	[13]
		ScB (2.5, 5, and 10 M) for 30 min and then exposed to CA (10 M) for 24 h	LDH release Cell viability Cellular apoptosis GSH and ATP levels ROS levels	ScB ↓ LDH release and ↑ cell viability ScB ↓ number of early apoptotic cells ScB ↑ GSH and ATP levels	[41]
	KM adult male mice	Four groups Control: olive oil (10 mL/kg, gavage) and then olive oil (2 mL/kg, SC) ScB: 20 mg/kg, gavage, and then olive oil (2 mL/kg, SC) CA: olive oil (10 mL/kg, gavage), and then CA (30 mg/kg, SC) CA+ScB: ScB (20 mg/kg, gavage), and then CA (30 mg/kg, SC) Treatment duration: 28 days	LDH release Cellular apoptosis GSH and ATP levels ROS levels GSH level MDA level	↓ BUN and creatinine levels ↑ GSH and ↓ MDA levels ScB markedly improved the structural changes induced by CA	[41]
Lycopene	Male Swiss albino rats	Four groups Control: saline solution, I.P. Lyc: 40 mg/kg/day, oral gavage CA: 15 mg/kg/day, I.P. for 10 days LYC + CA: LYC (40 mg/kg/day, oral gavage for 5 days then CA (15 mg/kg/day, I.P. for 10 days) concomitantly with LYC Treatment duration: 15 days	Serum creatinine and urea levels MDA level GSH-Px activity SOD activity	LYC ↓ urea and creatinine levels LYC restored GSH and ↓ MDA levels LYC ↑ Gpx and SOD activities LYC markedly improved CA-induced structural changes	[26]
	Adult male Sprague Dawley rats	Four groups Control: 0.5 mL isotonic saline + 0.5 mL corn oil, SC for 21 days CA: 15 mg/kg/day CA + 0.5 mL corn oil, SC for 21 days LYC: 10 mg/kg/day LYC + 0.5 mL corn oil, SC for 21 days CA+LYC: 10 mg/kg/day LYC + 15 mg/kg/day CA, SC for 21 days Treatment duration: 21 days	TBARs level GSH level GSH-Px activity CAT activity Plasma creatinine, urea, Na^+^ and Ca^++^	LYC ↓ plasma creatinine and urea levels LYC normalized TBARs LYC ↑ GSH-Px and CAT activity LYC alleviated CA-induced histological changes	[42]
S-Allylcysteine (SAC)	Wistar male albino rats	Five groups Control: oral saline CA: 25 mg/kg/day, orally CA+SAC: CA 25 mg/kg b.w./day with SAC 100 mg/kg/day, I.P. CA+ Vit. C: 25 mg/kg/day with vitamin C 100 mg/kg/day, orally SAC: 100 mg/kg/day I.P. Treatment duration: 21 days	Serum renal markers urea, uric acid, creatinine, and BUN Renal enzymes (ALP, ACP, AST, ALT, and LDH) Antioxidants enzymic (SOD, CAT, GPx, and GR) non-enzymic (GSH, vits C and E) Expressions of NF-kB, iNOS, and MMP-2	SAC prevented alteration in urea, uric acid, creatinine, and BUN induced by CA the same as vit C SAC ↓ the rise in LPO induced by CA compared with vit. C SAC ↑ antioxidants levels compared with vit. C SAC ↓ renal injury and inflammation SAC suppressed the expressions of NF-kB, MMP-2, and iNOS	[27]
Curcumin	Adult male rats	Three groups Control Curcumin+CA (15 mg/kg with CA) CA: 20 mg/kg, SC/5 days Treatment duration: 21 days	Morphological changes Glutathione S-transferase (GST) immune expression Serum urea and creatinine	Curcumin showed promising protective effect against CA-induced nephrotoxicity in rats through improving histological parameters, antioxidant effect, and renal dysfunction	[33,43,44]
		Five groups Vehicle: olive oil SC + 0.5% CMC orally for 21 days CA: 20 mg/kg/day, SC in olive oil for 21 days CA+ Curcumin: CA (20 mg/kg/day SC) + Curcumin (5, 10, or 15 mg/kg for 21 days Treatment duration: 21 days	Oxidative stress in kidney tissues (TBARS, GSH, SOD, and CAT)	Curcumin ameliorated renal dysfunction through decreasing TBARS and increasing levels of antioxidant enzymes (GSH, SOD, and CAT)	[45]
Resveratrol (RES)	Male albino Wistar rats	Eight groups Control Vehicle: olive oil (SC) + saline (p.o.) for 21 days CA: CA (20 mg/kg, SC) in olive oil for 21 days RES 2 mg+CA: RES (2 mg/kg, p.o.) 24 h before CA and continued along with CA for 21 days RES 5 mg+CA: RES (5 mg/kg, p.o.) 24 h before CA and continued along with CA for 21 days RES 10 mg+CA: RES (5 mg/kg, p.o.) 24 h before CA and continued along with CA for 21 days RES+ L-NAME: RES (5 mg/kg, p.o.) and L-NAME (10 mg/kg, I.P.) 24 h before CA and continued along with CsA for 21 days L-NAME: L-NAME (10 mg/kg, I.P.) 24 h before CA and continued along with CA for 21 days Treatment duration: 21 days	Renal oxidative stress Renal functions Tissue and urine nitrite and nitrate levels Renal lipid peroxides and antioxidant enzymes Renal histology	RES (5, 10 mg/kg) improved renal dysfunction, renal and tissue NO levels, and oxidative stress Co-administration of L-NAME blocked RES protective effect indicating that RES effect is NO-dependent	[37]
Naringin (NG)	Male albino Wistar rats	Four groups Control: olive oil CA: CA 25 mg/kg for 21 days NG: NG 40 mg/kg/orally CA+NG: CA-treated group concurrently with NG daily (oral dose 40 mg/kg) Treatment duration: 21 days	Lipid peroxides (TBARS) and hydroxyl radical (OH) SOD, CAT, GSH, and vitamins C, E, and A	NG significantly ↓ oxidative stress and restored the levels of enzymic and non-enzymic antioxidants in renal tissues	[29]
Caffeic acid phenethyl ester (CAPE)	Wistar albino female rats	Four groups Control: 0.5 mL normal saline CA: CA (15 mg/kg/day SC in saline for 10 days) CAPE: CAPE 10 µM/kg/day, I.P. in saline for 11 days CAPE+CA: CAPE 10 µM/kg/day, I.P. for 11 days, while CA was administered concurrently for 10 days Treatment duration: 11 days	MPO activity Lipid peroxidation SOD and CAT	CAPE prevented increase in MDA CAPE ↑ CAT, but did not affect MPO and SOD	[36]
Catechin (CATC)	Wistar rats (both sexes)	Five groups Control: olive oil (S.C) and 0.5% sodium CMC orally CA: CA (20 mg/Kg/day/SC, for 21 days) CA+50mg CATC: CA (20 mg/Kg/day, SC)+ CATC (50 mg/kg/day P.O) for 21 days CA+100mg catechin: CA (20 mg/Kg/day, SC)+ CATC (100 mg/kg/day, P.O) CATC: CATC (100 mg/kg/day, P.O.) Treatment duration: 11 days	Body weight, water intake, food intake, urine output and kidney weight, oxidative stress markers (renal MDA, glutathione, renal antioxidant enzymes, such as SOD, catalase)	CATC ↑ body weight by administration of CATC (100 mg/kg/day) with CA for 21 days CATC ↑ renal function by ↓ serum creatinine, blood urea nitrogen, and ↑ creatinine and urea clearance CATC (50 mg/kg/day) restored only increased serum creatinine levels	[22]
Epigallocatechin gallate PLGA NPs	Male Sprague Dawley rats	Six groups All receiving CA (15 mg/kg/day orally) and EGCG (50 mg/kg) Control Sandimmune neural (CA) SN+EGCG (I.P.) daily SN+EGCG (oral) daily SN+EGCG (I.P.) once in three days SN+EGCG (I.P.) once in three days (blank particles) Treatment duration: 30 days	BUN, PC, MDA, GSH, and total proteins Histological studies	↓ BUN and PC levels (I.P. injection of EGCG along with SN) Co-treatment of EGCG NPs as equally effective as I.P. administration EGCG solution prevented histological damage induced by SN treatment EGCG NPs prevented renal damage, artial glomerular collapse, and tubular damage	[38]
Ellagic acid	Adult male Sprague Dawley rats	Four groups Control: 0.5 mL isotonic saline+ 0.5 mL slightly alkaline solution, SC for 21 days CA: 15 mg/kg+0.5 mL slightly alkaline solution S.C for 21 days Ellagic acid: 0.5 mL isotonic saline+10 mg/kg ellagic acid SC for 21 days CA+ellagic acid, SC for 21 days Treatment duration: 21 days	MDA, GSH, GSH-Px, and CAT activities Histopathological examination	No significant change in GSH level Slight decrease in MDA level Ellagic acid ↑ decreased GSH-Px activity and CAT activity due to CA intake Ameliorated tubular necrosis, tubular degeneration, and desquamation, thickening basement membrane, inter-tubular haemorrhagia, and tubular dilatation	[46]
Silibinin	Female Wistar rats	Four groups Control: standard chow and I.P. injection of placebo solution Silibinin: standard chow and I.P. silibinin 5 mg/kg CA: 30 mg/kg I.P. CA+Silibinin: I.P. silibinin and CA Treatment duration: 15 days	Urea, creatinine, total protein, and GFR Lipid peroxidation Histopathological examination Total MDA	Silibinin ↓ CA-induced LPO without protective effect on GFR	[47]
Naringenin (NGA) and VOO		Four groups Control: saline CA: 25 mg/kg/day for 45 days CA+NGA: NGA 100 mg/kg/day+ CA 25 mg/kg/day for 45 days CA+VOO: VOO 1.25 mL/kg/day + CA 25 mg/kg/day for 45 days Treatment duration: 45 days	Body and kidney weights Cyclosporine blood level Renal biochemical markers Redox status	VOO and NGA ↑ body and kidney weights VOO and NGA ↓ CA blood level VOO and NAR ↓ serum creatinine and urea levels VOO and NGA ↑ CAT, peroxidase, GSH, and SOD VOO and NGA ↓ MDA and NO	[28]
Provinol (PV) mixture of polyphenolics: proanthocyanidins, total anthocyanins, free anthocyanins, catechin, hydroxycinnamic acid, and flavonols	Adult male Wistar rats	Four groups Control: olive oil, SC PV: PV (40 mg/kg/day orally) CA: CA (15 mg/kg/day/SC) PV+CA: PV and CA simultaneously Treatment duration: 21 days	Systolic blood pressure Creatinine clearance Oxidative stress NF-kB NO-synthase	PV ↓ oxidative stress and ↑ iNOS and NF-kB expression induced by CA	[48]
	Ninety-day-old virgin female and male Wistar rats	Four groups Control: olive oil, SC PV: PV (40 mg/kg/day/orally) CA: CA (15 mg/kg/day/SC) CA+PV: CA and PV I.P. daily for 21 days of pregnancy Treatment duration: 21 days	Histopathological and immunohistochemical evaluations Stereological analysis for glomerular volume and size iNOS and MMP2	PV ↓ oxidative stress and iNOS expression via NF-kB pathway PV protected on CA-induced structural and functional alterations of the kidney	[49]
	Adult male Wistar rats	Four groups Control: olive oil (SC) CA: CA (15 mg/kg/day/ SC) for 21 days PV: (40 mg/kg/day, orally) PV+CA: PV concurrently during CA injections for 21 days Treatment duration: 21 days	Body weight, SBP, serum creatinine, urinary protein concentration, GFR, creatinine clearance, renal GSH content	PV prevented CA-induced decrease in body weight and increase in SBP PV ↓ CA-induced depletion of GSH PV restored morphological and biochemical alterations	[50]

**Table 3:** Plants extracts and/or faction effects on CA-induced renal injury.

Name of Plant (Organ, Family)/Diet	Extract/Fraction (Major Constituents)	Intervention (Dose, Route/Administration Duration)	Studied Parameter	Pharmacological Outcomes/Effects	Reference
*Sargassum wightii* (Sargassaceae)	Sulphated polysaccharides (SWSPs)	Male albino Wistar rats Four groups Control: olive oil SWSP: SWSP (5 mg/kg/b.w., S.C.) SWSP+CA: SWSP (5 mg/Kg/b.w., SC)/3 weeks then CA (25 mg/kg/b.w., orally)/21 days CA: CA (25 mg/kg/b.w., orally/21 days) Treatment duration: 28 days	Glycosaminoglycans Protein content Creatinine clearance Lysosomal enzymes Serum and urinary creatinine Protein-bound carbohydrates	SWSP ↑ creatinine clearance SWSP ↓ lysosomal enzyme activity SWSP ↓ glycoproteins levels SWSP ↓ glycosaminoglycanuria SWSP ↓ proteinuria SWSP attenuated CA-induced histological changes	[69]
		Male albino Wistar rats Four groups Control: olive oil alone CA: 25 mg/kg/b.w., orally/21 days SWSP: 5 mg/Kg/b.w., SC SWSP+CA: SWSP (5 mg/Kg/b.w., SC)/3 week then CA (25 mg/kg/b.w., orally)/21 days. Treatment duration: 28 days	Enzymes (ALP, NAG, LDH, and c-GT) Mitochondrial oxidative stress (ROS, SOD, GPx, and GSH) Lipid peroxidation (MDA) TCA cycle enzymes Enzyme complexes of electron transport chain	SWSP ↓ ROS level and lipid peroxidation SWSP ↑ antioxidant defense system SWSP ↑ activities of tricarboxylic acid cycle and electron transport chain enzymes and ↓ urinary enzymes SWSP ameliorated mitochondrial swelling	[68]
Dietary fish oil (DFO)		White male albino rats Four groups Control: no drugs DFO: DFO (270 mg/kg/day, orally) CA: CA (25 mg/kg/day orally for 21 days) DFO+CA: DFO for 21 days before, 21 days concurrently during CA and 21 days later Treatment duration: 63 days	Serum glucose, TP, albumin, and lipid profile (TC, TAGs, and PLs) Renal function (urea, UA and Cr, and electrolytes (Na and K)) Inorganic phosphorus and haptoglobin levels LDH and GGT activities MDA, GSH, NO, and TOA levels CAT, SOD, and Gpx activities	DFO restored urea, uric acid, creatinine, and haptoglobin levels DFO normalized lipid profile, LDH, CGT, serum protein, and electrolytes DFO ↓ peroxidative levels and ↑ CAT, SOD, and GPx activities DFO ↑ GSH and TAC levels	[40]
Probiotic formulation LOBUN		Male Wistar rats Four groups Normal: olive oil Control: CA (20 mg/kg/day, SC) for 15 days CA+LOBUN: CA (20 mg/kg/day, SC) for 15 days; LOBUN (500 mg/kg/p.o./twice daily) from 15th to 28th day CA+LOBUN: CA (20 mg/kg/day, SC) for 15 days; LOBUN (500 mg/kg p.o./thrice daily) from 15th to 28th day Treatment duration: 28 days	BUN, serum Cr, serum UA, total serum and urine proteins, and urine K and Na	LOBUN ↓ serum BUN, serum Cr, serum UA and ↑ total serum protein LOBUN ↓ urine protein concentration and ↑ Na and K LOBUN ↓ inflammatory infiltration and vacuolization	[80]
* Zingiber officinale * (Zingebraceae)	80% rhizome acetone extract rich in polyphenols (GP)	Male Wistar rats Five groups Control: distilled H_2_O CA: CA (50 mg/kg, p.o.) for 10 consecutive days CA+GP: CA (50 mg/kg, p.o.) + GP (100 mg/kg, p.o.) for 21 days CA+GP: CA (50 mg/kg, p.o.) +GP (200 mg/kg, p.o.) for 21 days CA+GP: CA (50 mg/kg, p.o.) + GP (400 mg/kg, p.o.) for 21 days Treatment duration: 21 days	Percentage of body weight Kidney weight changes Food consumption, water intake, and urine volume Creatinine clearance Electrolyte assays Oxidative stress	GP attenuated kidney injury caused by CA through improvement of plasma and urine levels of creatinine, urea, Na+ and K+ electrolyte balance, as well as creatinine clearance GP improved feeding pattern, relative kidney weight, and oxidative stress	[76]
Date pits (DPEs)	Aqueous extract	Male Wister albino rats Four groups Control: 0.5 mL of NaCl/ day for 28 days CA: CA (15 mg/kg/day, SC) for 28 days DPE+CA: DPE (4 mL/ kg/ day)+CA (15 mg/kg/d) for 28 days DPE+CA: DPE (6 mL/ kg/ day, orally)+CA (15 mg/kg/ day) for 28 days Treatment duration: 28 days	Serum Cr, BUN, UA, sodium, potassium, total protein, and albumin levels MDA, GSH, and CAT activities Histological changes	DPE ameliorated all measured parameters DPE protected against CA-induced histopathological changes DPE ↓ MDA and ↑ GSH and CAT activities	[77]
*Spinacea oleracea*	1%*n*-hexane extract (SOH)	Rats Four groups Control: olive oil (2 mL/kg, I.P., 7 days) CA: CA (20 mg/kg in 2 mL olive oil I.P., 7 days) SOH+CA: SOH (0.5 mL concomitantly with CA (20 mg/kg in 2 mL olive oil, I.P.) from days 7 to 14 SOH: SOH (0.5 mL orally for 14 days) Treatment duration: 14 days	Histological changes	SOH significantly restored all disturbed histologic parameters SOH ↓ glomerular diameter	[78]
*Ginseng*	Korean red ginseng extract (KRG)	Mice Eight groups VH: olive oil (5 mL/kg, SC daily) and oral sterile water for 4 weeks VH+KRG 0.2: Olive oil (5 mL/kg, SC) + KRG (0.2 g/kg, orally) for 4 weeks VH+KRG 0.4: Olive oil (5 mL/kg, SC) + KRG (0.4 g/kg, orally) for 4 weeks VH+KRG 0.8: Olive oil (5 mL/kg, SC) + KRG (0.8 g/kg, orally) for 4 weeks CA: CA (30 mg/kg, SC) and oral sterile water for 4 weeks CA+KRG 0.2: CA (30 mg/kg, SC) + KRG (0.2 g/kg, orally) for 4 weeks CA+KRG 0.4: CA (30 mg/kg, SC) + KRG (0.4 g/kg, orally) for 4 weeks CA+KRG 0.8: CA (30 mg/kg, SC) + KRG (0.8 g/kg, orally) for 4 weeks Treatment duration: 28 days	Assessing renal function and pathology, mediators of inflammation, tubulointerstitial fibrosis, and apoptotic cell death Effect on proximal tubular cells (HK-2) by in vitro model Assessing 8-OHdG levels in 24 h urine, tissue sections, and culture media	KRG ↓ serum Cr and BUN KRG ↑Cr clearance KRG ↓ proinflammatory and profibrotic molecules as iNOs, cytokines, TGF-β1, and TGF-β1-inducible gene h3 and apoptotic cell death In vitro studies, KRG ↑ protection against CA-induced morphological changes, cytotoxicity, inflammation, and apoptotic cell death KRG ↓ 8-OHdG level in urine and culture supernatant	[79]
Black grape/garlic extract	Dried fruit (BG)/aqueous extract (GAE)	Sprague Dawley rats Six groups CA: CA (25 mg/kg/day, orally) for 10 days, with food supplementation (3 days before CA treatment and continued during the study period (13 days)) Control CA: CA (25 mg/kg/day, orally) for 10 days CA+ BG: BG (25 g/kg/day with diet) CA+GAE: GAE (20 mL/kg/day with drinking H_2_O) BG: BG (25 g/kg/day with diet) GAE: GAE (20 mL/kg/day with drinking H_2_O) Treatment duration: 13 days	Oxidant (XO and MDA) and antioxidant (SOD, GSH-Px, and CAT) enzymes Histopathological changes	BG and GAE produced no changes in XO, SOD, and GSH-Px activities BG and GAE ↓ MDA level and CAT activity BG and GAE ameliorated glomerular sclerosis tubular necrosis and interstitial fibrosis	[81]
Green tea	Lyophilized aqueous extract (GTE)	White male albino rats Four groups Control GTE: GTE (3 %W/V) for 9 weeks CA: CA (25 mg/kg/orally/day) for 21 days GTE+CA: GTAE (3% W/V) for 21 days before CA, then for 21 days concomitant with CA followed by 21 days later Treatment duration: 63 days	Serum glucose, TP, albumin, TC, TAGs, PLs, urea, UA, Cr, Na, K, inorganic phosphorus, LDH, GGT. MDA, GSH, NO, TAO, CAT, SOD, and GPX	GTE improve renal function and ↓ peroxidative levels GTE ↑ renal tissues antioxidant by enhancing CAT, SOD, GPX, and TAC activities GTE restored elevated glucose, lipid profile, urea, UA, Cr, LDH, and GGT. GTE reversed ↑ in serum proteins and electrolyte to normal range	[82]
	Lyophilized aqueous extract	Sprague Dawley rats Six groups Control CA: CA (20 mg/kg/day, I.P.) for 21 days GTE0.5+CA: GTE (0.5% with drinking water, 4 days before and 21 days concurrently with CA GTE0.5+CA: GTE (1% with drinking water, 4 days before and 21 days concurrently with CA GTE0.5+CA: GTE (1.5% with drinking water, 4 days before and 21 days concurrently with CA GTE: GTE (1.5% with water) for 25 days Treatment duration: 21 days	Glucose, Cr, BUN, serum UA, and Cr levels GSH and TBARS Enzyme activities: CAT, SOD, GPx, GR, GST, NAG, β-GU, and AP	GTE prevented TBARS regeneration GTE ↓ CA-induced renal dysfunction as indicated by ↓ serum Cr, BUN, UA, and urinary excretion of glucose GTE ↑ reduced glutathione content and activity of antioxidant enzymes in the kidney homogenate GTE ↓ activity of lysosomal enzymes; NAG and AP	[83]
Propolis	Ethanol extract	Sprague Dawley rats Four groups Control: no supplement CA: CA (15 mg/kg/day SC) Propolis (100 mg/kg/day, gavage) CA+Propolis: CA (15 mg/kg/day SC) + Propolis (100 mg/kg/day, gavage) Treatment duration: 21 days	Serum cortisol, glucose, albumin, globulin, TP, urea, TAGs, HDL, VLDL, LDL, TC, Cr, AST, and ALT values	Propolis ↓ improved CA-induced BW reduction Propolis ↓ cortisol, AST, ALT, urea, and MDA levels in kidney Propolis ↑ CAT and GSH activities	[84]
Spirulina (algae, *Arthrospira platensis)*		Male Sprague Dawley rats Eight groups Control R: single dose of whole-body gamma irradiation (6.5 Gy) CA: CA (25 mg/kg, I.P.) for 10 days CA+R: CA for 10 days, then exposed to gamma radiation on the last day Sp: Sp (1 g/kg, intragastric gavages for 15 consecutive days) Sp+R: Sp for 15 days before irradiation Sp+CA: Sp for 5 days before and 10 days concomitant with CA Sp+CA+R: Sp for 5 days before and 10 days concomitant with CA injection and exposed to gamma radiation Treatment duration: 15 days	Serum creatinine, urea, glucose, albumin, protein, and lipid profile as well as GSH, TBARS, nitrite, and SOD activities Trace elements (Zn and Mg) Caspase-3 expression Histopathological changes	Sp ↓ serum creatinine, urea, and glucose levels of CA-administrated rats Treatment of irradiated CA- administrated rats with Sp ↓ serum creatinine and urea Sp ↓ serum albumin and protein levels of R group (20 and 17%, respectively), CA group (20 and 13%, respectively), and CA + R group (28 and 21%, respectively) Sp ↓ Zn and ↑ Mg content of kidney	[85]
*Cordyceps cynensis* (CS)	Pharmaceutical product	Concurrent administration of *Cordyceps sinensis* in CA-treated kidney-transplanted recipients. Each recipient was given CA (5 mg/kg/day for 15 days) Control: placebo (glucose) 3 g CS: *Cordyceps sinensis* 3g simultaneously Treatment duration: 15 days	Blood creatinine, urea, and NAG	SC ↓ creatinine, urea, and NAG SC protected the proximal tubular function and ameliorated renal hemodynamics	[86]
Fennel, carob, doum (FE, CA, DO)	Powdered FE seeds, CA pods, and DO fruit (17, 18, and 21 g/kg, respectively) were added to the experimental animal’s diet	Female Sprague Dawley rats Six groups Control: injected with corn oil daily for 7 days CA: CA (50 mg/kg/day in corn oil for 1 week) CA+FE, CA, and DO: injected CA for 7 days then FE, CA, and DO and mixture of them was added to the diet of these groups, respectively Treatment duration: 45 days	Creatinine levels in serum and urinary samples, serum ammonia, TGF-β, TNFα, NAG, and β2MG Histopathological examination	FE, CA, and DO mixture ↓ serum creatinine, urinary creatinine, and serum ammonia levels They ↑ creatinine clearance They ↓ urinary β2MG and NAG activity and ↓ levels of serum TNF-α and TGF-β They significantly ameliorated functions and morphological structure of the kidney	[87]
*Ipomea batates* (LB)	Aqueous extract	Male rats Four groups Control: distilled water for 2 weeks, then olive oil, I.P. for 21 days CA: distilled water for 2 weeks, then CA (25 mg/kg, I.P. in olive oil/21 days) LB200 + CA: LB 200 mg/kg, orally) for 21 days, then CA LB400 + CA: LB 400 mg/kg, orally) for 21 days, then CA Treatment duration: 21 days	Oxidative stress biomarkers (MDA and SOD) Cytokines (IL-1β and TNF-α) Kidney function (BUN, UA, Cr) Na+ and K+serum levels Histopathological studies	LB ↓MDA and ↑SOD activity LB ↓ TNF-α and IL1-β LB ↓ BUN, UA, and Cr LB ↑ ionic Na+ level and ↑ ionic K+ level	[88]
*Nigella sativa* oil (NSO)	Fixed oil	Male Wistar albino rats Four groups Control: sunflower oil (2 mL/kg/day, orally) NSO: NSO (2 mL/kg orally/21 days) CA: CA (25 mg/kg, orally/21 days) CA + NSO: NSO (2 mL/kg orally) since the first day, while CA (25 mg/kg orally) for the last 21 days Treatment duration: 21 days	Urine and serum Cr levels Total (Cu-Zn, Mn) SOD activities CAT, GSH-Px, and MDA Kidney nitrite and nitrate levels	No significant amelioration of Cr levels and SOD activities for groups CA with NSO NSO ↑ GSH-Px level NSO ↓ MDA and NO levels	[89]
*Schisandrae chinensis* (SCE)	Fruit 95% alcohol extract	Male Sprague Dawley rats Seven groups Vehicle: olive oil (10 mL/kg) for 14 days CA: CA (50 mg/kg) for 3 days CA + SCE: 50 mg/kg CA + 216 mg/kg SCE for 3 days CA group: 50 mg/kg CA for 7 days CA + SCE: 50 mg/kg CA + 216 mg/kg SCE for 7 days CA: 50 mg/kg CA for 14 days CA + SCE: 50 mg/kg CA + 216 mg/kg SCE for 14 days Treatment duration: 14 days	Cre and BUN GSH, CAT, MDA, and SOD Pathologic manifestations	SCE ↓ CRE and BUN levels SCE ↑ GSH, CAT, and SOD and ↓ MDA In the 14-day group, no glomerular balloon occlusions or vacuolar lesions were observed, and the tissues presented good renal characteristics	[90]

## Data Availability

Not applicable.

## Abbreviations

2-DG: 2-Deoxy-D-glucose; 8-iso-PGF2α: 8-epi-prostaglandin F2α; ALP: alkaline phosphatase; AP: acid phosphatase; 8-OHdG: 8-hydroxy-2′-deoxyguanosine; AREs: antioxidant-response elements; b.w.: body weight; BUN: blood urea nitrogen; CA: cyclosporine A; CAT: catalase; c-GT; c-Glutamyl transferase; CVD: cardiovascular disease; CKD: chronic kidney disease; CMC: carboxy methyl cellulose; Cr: creatinine; CYP3A: cytochrome P450, family 3, subfamily A; EGCG: epigallocatechin gallate; EMT: epithelial–mesenchymal transition; GCLM: glutamate-cysteine ligase-modifier subunit; GFR: glomerular filtration rate; GGT: gamma glutamyl transferase; GL: gastric lavage; GSH-Px: glutathione peroxidase; GR: glutathione reductase; GSH: reduced glutathione; GST: glutathione-*S*-transferase; HK-2: human proximal tubular epithelial cell line; HO-1: heme oxygenase-1; HSP-70: heat shock protein-70; I.P.: intra-peritoneal; iNOS: inducible nitric oxide synthase; LDH: lactate dehydrogenase; c-GT: c-Glutamyl transferase; LDL: low-density lipoprotein; LPO: lipid peroxidation; MMP-2: matrix metalloproteinase-2; MetS: metabolic syndrome; MUFAs: monounsaturated fatty acids; NAG: *N*-acetyl-β-d-glucosaminidase; NF-kB: nuclear factor kappa B; NO: nitric oxide; NPs: nanoparticles; NQO1 NAD(P) H: quinone oxidoreductase 1; Nrf2: nuclear factor erythroid 2-related factor 2; OHdG: 8-Hydroxy-2′-deoxyguanosine; OS: oxidative stress; p.o.: perorally; PC: plasma creatinine; PLs: phospholipids; PLGA: poly(lactic-co-glycolic) acid; Px: peroxidase; ROS: reactive oxygen species; RPF: renal plasma flow; SC: subcutaneously; SBP: systolic blood pressure; ScB: Schisandrin B; SWSPs: Sargassum wightii-sulphated polysaccharides; TAO: total antioxidant capacity; TBARS: thiobarbituric acid-reactive substances; TC: total cholesterol; TAGs: triacylglycerols; TP: total protein; TX: thromboxane; TGF-β1: transforming growth factor-β1; TQ: thymoquinone; UA: uric acid; VOO: virgin olive oil; XO: xanthine oxidase; β2MG: β2-microglobulin; β-GAL: β-galactosidase; ΔΨm: mitochondrial transmembrane potential.
